# Heart rate variability during infusion of dexmedetomidine

**DOI:** 10.1186/cc9772

**Published:** 2011-03-11

**Authors:** T Imabayashi, K Ikoma, T Kikuchi, Y Kakihana, Y Kanmura

**Affiliations:** 1 Kagoshima University Hospital, Kagoshima, Japan

## Introduction

Dexmedetomidine is an α_2_-agonist, used for sedation in the ICU, although much remains to be learned about the effects on the autonomic nervous function. We therefore investigated them in the real-time monitoring of heart rate variabilities.

## Methods

From May through November 2010, 20 patients were selected if they were treated on total mechanical ventilatory support and they were treated with continuous infusion of dexmedetomidine in our ICU. The exclusion cases were with arrhythmia or pacemaker or other treatment during the measure time. Heart rate (HR) variability analysis was recorded using the MemCalc system (MemCalc/Tonam16C; Suwa Trust, Tokyo, Japan). The spectral bands were 0.04 to 0.15 Hz (low frequency (LF)), 0.15 to 0.40 (high frequency (HF)) and others. The HF component was an indicator of sympathetic balance, and LF/HF was that of parasympathetic balance. We measured the HR, CV-RR, HF, LF/HF, systemic blood pressure (SBP), CV-SBP, SBP-HF and SBP-LF/HF. The CV-RR was SD of RR intervals, and the CV-SBP was SD of systemic blood pressure. We compared them between before and after 30 minutes administration of dexmedetomidine. The Wilcoxon signed-ranks test was used to compare the differences. *P *< 0.05 was considered statistically significant.

## Results

The HR was significantly decreased (*P *= 0.017), and the CV-RR was in a tendency of decrease (*P *= 0.085). Although the SBP was not significantly changed, the CV-SBP was significantly decreased (*P *= 0.038). Other parameters (HF, LF/HF, SBP-HF and SBP-LF/HF) were not significantly changed.

## Conclusions

We investigated the autonomic nervous functions in 20 patients treated with dexmedetomidine. The HR and the CV-SBP were significantly decreased. Dexmedetomidine was affected with depression of sympathetic nerve system to the HR, the CV-RR and CV-SBP.
